# MMPCS: multi-view molecular pretraining based on consistency information and specific information

**DOI:** 10.1093/bioinformatics/btag028

**Published:** 2026-01-14

**Authors:** Chenyang Xie, Yingying Song, Song He, Xiaochen Bo, Zhongnan Zhang

**Affiliations:** School of Informatics, Xiamen University, Xiamen 361005, China; School of Informatics, Xiamen University, Xiamen 361005, China; Department of Advanced & Interdisciplinary Biotechnology, Academy of Military Medical Sciences, Beijing 100850, China; Department of Advanced & Interdisciplinary Biotechnology, Academy of Military Medical Sciences, Beijing 100850, China; School of Informatics, Xiamen University, Xiamen 361005, China

## Abstract

**Motivation:**

The goal of molecular representation learning is to automate the extraction of molecular features, a critical task in cheminformatics and drug discovery. While pretraining models using multiple views like SMILES, 2D graphs, and 3D conformations have advanced the field, integrating them effectively to produce superior representations remains a challenge.

**Results:**

To bridge this gap, we propose a novel multi-view molecular pretraining method termed MMPCS, which explicitly factorizes representations into consistency and specific information. Our approach utilizes the Graph Isomorphism Network and the RoBERTa model to encode 2D molecular topological graphs and SMILES sequences, respectively. Each resulting molecular embedding is decomposed into a shared consistency component and a view-specific remainder. An autoencoder then aligns the consistency information across views. The combined consistency and view-specific representations serve as input for downstream tasks, enabling precise and task-aware predictions. When benchmarked against 16 state-of-the-art molecular pretraining methods, MMPCS achieved the highest average performance across both classification and regression tasks for molecular property prediction. It also delivered outstanding results in predicting drug-target binding affinity and cancer drug response, demonstrating its robustness and broad applicability. Additionally, a case study on the SARS-CoV-2 Omicron variant highlights the potential of MMPCS in facilitating drug repurposing efforts.

**Availability and implementation:**

The source code and datasets supporting this study are publicly available at GitHub (https://github.com/xmubiocode/MMPCS) and Zenodo (https://doi.org/10.5281/zenodo.18182748).

## 1 Introduction

Molecular representation learning focuses on the automatic extraction of molecular features and plays a critical role in various downstream tasks such as molecular property prediction (Yang *et al.* 2019) and molecular design (Du *et al.* 2022). Deep learning has been widely adopted in this field, significantly enhancing the efficiency of molecular representation learning, and driving advancements in cheminformatics and drug discovery. However, deep-learning models typically rely on large amounts of labeled data for effective training. In data-scarce scenarios, these models are prone to overfitting and may capture spurious correlations (Sagawa *et al.* 2020). To maximize the potential of deep learning in molecular representation learning, addressing the challenges posed by insufficient labeled data is essential.

In recent years, inspired by the remarkable success of leveraging large-scale unlabeled data in natural language processing and computer vision, pretraining has been increasingly applied to molecular representation learning (Xia *et al.* 2023a). These models are typically pretrained on objectives derived from extensive unlabeled datasets and subsequently fine-tuned on labeled downstream tasks.

Pretrained models in the molecular domain can be broadly categorized into three classes by input modalities. The first category relies on Simplified Molecular Input Line Entry System (SMILES) sequences (Weininger 1988). Early approaches, such as SMILES-Transformer (Honda *et al.* 2019), adopted autoencoder architectures (Hinton and Salakhutdinov 2006) that trained the decoder to reconstruct the original input sequence. Subsequent approaches, such as SMILES-BERT (Wang *et al.* 2019) and ChemBERTa (Ahmad *et al.* 2022), introduced masked language modeling by randomly masking parts of the SMILES sequences during training. Recent studies have further enhanced SMILES-based pretraining by incorporating strategies such as functional-group-level masking (Peng *et al.* 2025) and randomized SMILES augmentation (Lee *et al.* 2025). Despite these advancements, these models are inherently limited by their reliance on linear representations, which struggle to capture the intrinsic structural information of molecules.

Pretraining models based on 2D molecular topological graphs represent the second category of approaches. Hu *et al.* (Hu *et al.* 2020) introduced masked graph modeling by predicting masked node attributes or masked substructures. GraphMAE (Hou *et al.* 2022), on the other hand, reconstructed masked node features using a decoder. Beyond masking strategies, contrastive learning has been widely adopted. Methods such as GraphCL (You *et al.* 2020), JOAOv2 (You *et al.* 2021), AD-GCL (Suresh *et al.* 2021), and MolCLR (Wang *et al.* 2022) learned by maximizing the consistency between augmented graph views. Other studies have further extend molecular representation learning through motif-level self-supervision (Yan *et al.* 2024) or subgraph-conditioned bottlenecks for core-structure disentanglement (Hoang and Lee 2025). Common graph augmentations include node dropout, edge perturbation, feature masking, and subgraph extraction. However, even minor structural perturbations may distort molecular semantics. To mitigate this issue, MoCL (Sun *et al.* 2021) incorporated domain knowledge to guide substructure replacement, but this limits its generality and increases costs. Such methods that rely heavily on data augmentation have limitations in terms of efficiency and applicability. To avoid semantic distortion and reduce dependence on data augmentation, our model entirely eliminates the use of data augmentation.

The third category comprises pretrained models based upon multiple molecular views. Multi-view approaches have demonstrated significant advantages in bioinformatics for learning richer molecular representations. For instance, GraphMVP (Liu *et al.* 2022) jointly utilized 2D molecular graphs and computationally expensive 3D geometries. MM-Deacon (Guo *et al.* 2022) leveraged both SMILES and IUPAC sequences, while DVMP (Zhu *et al.* 2023) applied contrastive learning between SMILES and 2D graphs. MEMO (Zhu *et al.* 2022) integrated four distinct modalities: SMILES sequences, 2D graphs, 3D structures, and molecular fingerprints (Cereto-Massagué *et al.* 2015). By integrating 2D, 3D, and textual data, Uni-Mol2 (Wang *et al.* 2024) introduced a unified multi-view framework for molecular representation However, existing multi-view approaches predominantly emphasize cross-view consistency, often overlooking the view-specific information that not only complemented such consistency but also provides additional discriminative cues. Fully leveraging both consistency and view-specific information can yield more comprehensive and expressive molecular representations (Chen *et al.* 2023).

To address this limitation, we introduce MMPCS, a multi-view molecular pretraining model that explicitly factorizes representations into consistency and view-specific information. Our model leverages two prevalent molecular representations: 2D topological graphs and SMILES sequences. Building on these two views, we design an innovative dual-view pretraining strategy. Frist, each view is fed into a dedicated encoder to extract its corresponding feature representation. The resulting embeddings are then decomposed into an inter-view consistency information shared across views and a view-specific information unique to each view. To ensure a clean disentanglement, we minimize the covariance between these two components. Subsequently, the consistency information across views is aligned through a mutual prediction task. For downstream tasks, the final molecular representation is formed by concatenating the aligned consistency information with the original specific information from each view, thereby providing a comprehensive encoding for enhanced predictive performance.

The main contributions of this study can be summarized as follows:

- We introduce a multi-view molecular pretraining model that incorporates not only the consistency information shared across different views but also the specific information unique to each view.
- MMPCS aligns consistency information across views without relying on data augmentation, thereby mitigating potential issues may arise from significant changes in molecular properties due to augmentation.
- MMPCS maintains high performance while requiring only a single copy of consistency information for downstream tasks, effectively reducing data dimensionality and improving computational efficiency.

To evaluate MMPCS, we began by pretraining the model on 500 000 randomly selected SMILES sequences from the ZINC dataset (Irwin and Shoichet 2005). Subsequently, MMPCS was fine-tuned on eight property classification datasets and five molecular property regression datasets for property prediction tasks. Compared with 16 classical and state-of-the-art methods, MMPCS achieved top or second-best performance on 11 molecular property prediction (MPP) datasets, and attained the highest average results across both the classification and regression tasks. Notably, MMPCS outperformed the most advanced multi-view pretraining models across all five regression datasets. Moreover, it achieved state-of-the-art performance in drug-target binding affinity (DTA) prediction and cancer drug response (CDR) tasks. Furthermore, visualization results show that MMPCS enhances the efficiency of molecular representations while preserving essential information. These findings collectively highlight the effectiveness and robustness of MMPCS in molecular representation learning.

## 2 Materials and methodology

### 2.1 Problem definition

Given a molecule M, let $M_{s}$ and $M_{g}$ denote the SMILES sequence and 2D topological graph of the molecule, respectively. $M_{s}$ is denoted by a sequence $\left(m_{1},\;m_{2},\;\cdots ,\;m_{l}\right)$, where l is its length. $M_{g}$ is represented as a graph $\left(V_{g},\;E_{g}\right)$, where $V_{g}={\{v}_{1},\;v_{2},\;\cdots ,\;v_{n}\}$ is the set of atoms constituting molecule M, and $E_{g}$ is the set of chemical bonds between atoms. The loss of the pretraining model can be expressed by Equation (1):


(#(1)) L(fθ(Ms, Mg))

where θ represents the parameters of the pretraining model f, and L(·) denotes the self-supervised learning loss function, which is a non-negative value. Our goal is to learn the optimal model parameters $\theta^{*}$ for f such that the loss is as close to zero as possible.

### 2.2 Datasets

The pretraining dataset comprises 500 000 randomly selected SMILES sequences from the ZINC (Table 1). For downstream tasks, we focused on MPP, CDR, DTA, and SARS-CoV-2 drug repurposing. To comprehensively evaluate the effectiveness of the proposed pretraining model, we used eight classification and five regression datasets, including six classification and three regression datasets from MoleculeNet (Wu *et al.* 2018). We further incorporated four additional datasets, Estrogen (Gaulton *et al.* 2012), MetStab (Podlewska and Kafel 2018), CEP (Hachmann *et al.* 2011), and Malaria (Gamo *et al.* 2010), to broaden the evaluation scope. The Estrogen and MetStab datasets offer biologically relevant information on pharmacological activity and metabolic stability, whereas CEP and Malaria extend the assessment to materials science and antimalarial screening, respectively. This diverse combination ensures MMPCS to be rigorously evaluated across a wide spectrum of chemical and biological properties. To ensure a rigorous and fair comparison with existing studies, we adopted different data splitting strategies. Following MoleculeNet’s recommendation, a scaffold-based split was applied to the BBBP (Martins *et al.* 2012) and BACE (Subramanian *et al.* 2016) datasets, which helps prevent structurally similar molecules from appearing across training and test sets, thereby offering a more challenging evaluation of model generalizability. For the remaining datasets, Tox21 (Huang *et al.* 2016), ToxCast (Richard *et al.* 2016), SIDER (Kuhn *et al.* 2016), ClinTox (Gayvert *et al.* 2016, Fromer and Coley 2023, Feng *et al.* 2024), Estrogen, MetStab, ESOL (Delaney 2004), Lipo, FreeSolv (Mobley and Guthrie 2014), Malaria, and CEP, we applied a conventional random split to maintain consistency with prior benchmark studies, as detailed in Tables 2 and 3. Together, these datasets cover key aspects of drug discovery, spanning pharmacology, physical chemistry, and physiology.

**Table 1:** Pretraining dataset information.

Property	Value
Molecules	500 000
Avg. atoms	27.4
Avg. bonds	29.8
Avg. degree	2.17
Avg. molecular weight	391.6

**Table 2:** Classification task datasets information.

Dataset	Compounds	Tasks	Split	Labeled data ratio
BBBP	2050	1	Scaffold	100%
Tox21	7831	12	Random	82.94%
ToxCast	8597	617	Random	27.92%
SIDER	1427	27	Random	100%
ClinTox	1484	2	Random	100%
BACE	1513	1	Scaffold	100%
Estrogen	3122	2	Random	63.57%
MetStab	2267	2	Random	100%

**Table 3:** Regression task datasets information.

Dataset	Compounds	Split
ESOL	1128	Random
Lipo	4200	Random
FreeSolv	642	Random
Malaria	9999	Random
CEP	29978	Random

For the CDR task, we used the commonly used GDSC dataset (Chapman *et al.* 2011). For the DTA task, we specifically utilized the KIBA dataset (Tang *et al.* 2014), an established benchmark in this domain. We followed its recommended data split method for reliable results. The model fine-tuned on KIBA was then directly applied to predict SARS-CoV-2 drug repurposing.

For models that require 2D molecular topological graphs as input, SMILES sequences were converted into molecular graphs using RDKit (Landrum 2006), and the features of nodes and edges were initialized to obtain 2D molecular topological graphs. SMILES sequences were converted into IUPAC identifiers via the STOUT model (Rajan *et al.* 2021) where such inputs were required.

### 2.3 Evaluation metrics

The pretraining dataset comprises 500 000 randomly selected SMILES sequences from the ZINC (Table 1). For downstream tasks, we focused on. To evaluate the performance of the model, the Receiver Operating Characteristic—Area Under the Curve (ROC-AUC) is used to evaluate the classification task in the downstream MPP task. ROC-AUC ranges from 0 to 1, with higher values indicating better performance. The ROC curve, plotting TPR against FPR, allows ROC-AUC computation via Equation (2):


(2) $$\mathrm{ROC}-\mathrm{AUC}=\int_{0}^{1}\mathrm{TPR}(\mathrm{FPR})\mathrm{dFPR}$$

The Root Mean Square Error (RMSE) is used to evaluate the regression task in the MPP. A smaller RMSE indicates that the model predictions ${\hat{y}}_{i}$ are closer to the true values $y_{i}$. The RMSE is expressed by Equation (3):


(3) $$\mathrm{RMSE}=\sqrt{\frac{1}{n}\sum_{i=1}^{n}({\hat{y}}_{i}-y_{i}{)}^{2}}$$

For the CDR task, the evaluation metrics are Pearson’s correlation coefficient (PCC) and Spearman’s correlation coefficient (SCC). PCC quantifies the linear relationship between two variables, on a scale from −1 to 1, based on the true values $y_{i}$, the predicted values $\hat{y_{i}}$, and their respective means $\bar{y}$ and $\overset{-}{\hat{y}}$. PCC can be expressed as follows:


(4) $$\text{PCC}=\frac{\sum_{i=1}^{n}\left(y_{i}-\bar{y})(\hat{y_{i}}-\overset{-}{\hat{y}}\right)}{\sqrt{\sum_{i=1}^{n}{\left(y_{i}-\bar{y}\right)}^{2}}\sqrt{\sum_{i=1}^{n}{\left(\hat{y_{i}}-\overset{-}{\hat{y}}\right)}^{2}}}$$

Unlike PCC, SCC measures the monotonic relationship between variables, does not assume linearity, and is robust to outliers. SCC can be expressed as follows:


(5) $$\text{SCC}=1-\frac{6\sum_{i=1}^{n}d_{i}^{2}}{n(n^{2}-1)}$$

where $d_{i}$ represents the difference in rank between the true and predicted values for sample i.

For the DTA task, Concordance Index (CI) and MSE are used for evaluation. CI measures the proportion of correctly ranked outcome pairs and is calculated as follows


(6) $$\text{CI}=\frac{\sum_{i<j}I(y_{i}<y_{j}\wedge \hat{y_{i}}<\hat{y_{j}})+0.5\cdot I(y_{i}=y_{j}\wedge \hat{y_{i}}=\hat{y_{j}})}{\sum_{i<j}I(y_{i}\neq y_{j})}$$

where I(⋅) is an indicator function that returns 1 if the condition is true and 0 otherwise. In addition, the quality of the molecular feature clusters is quantitatively assessed using the Davies–Bouldin Index (DBI), where a value closer to zero indicates better clustering performance. The formula for the DBI is as follows:


(7) $$\mathrm{DBI}=\frac{1}{k}\sum_{i=1}^{k}\underset{i\neq j}{\max}\left(\frac{R_{i}+R_{j}}{D_{ij}}\right)$$

where $R_{i}$ represents intra-cluster average distance and $D_{ij}$ refers to the inter-cluster center distance.

### 2.4 Method

**Figure 1:**
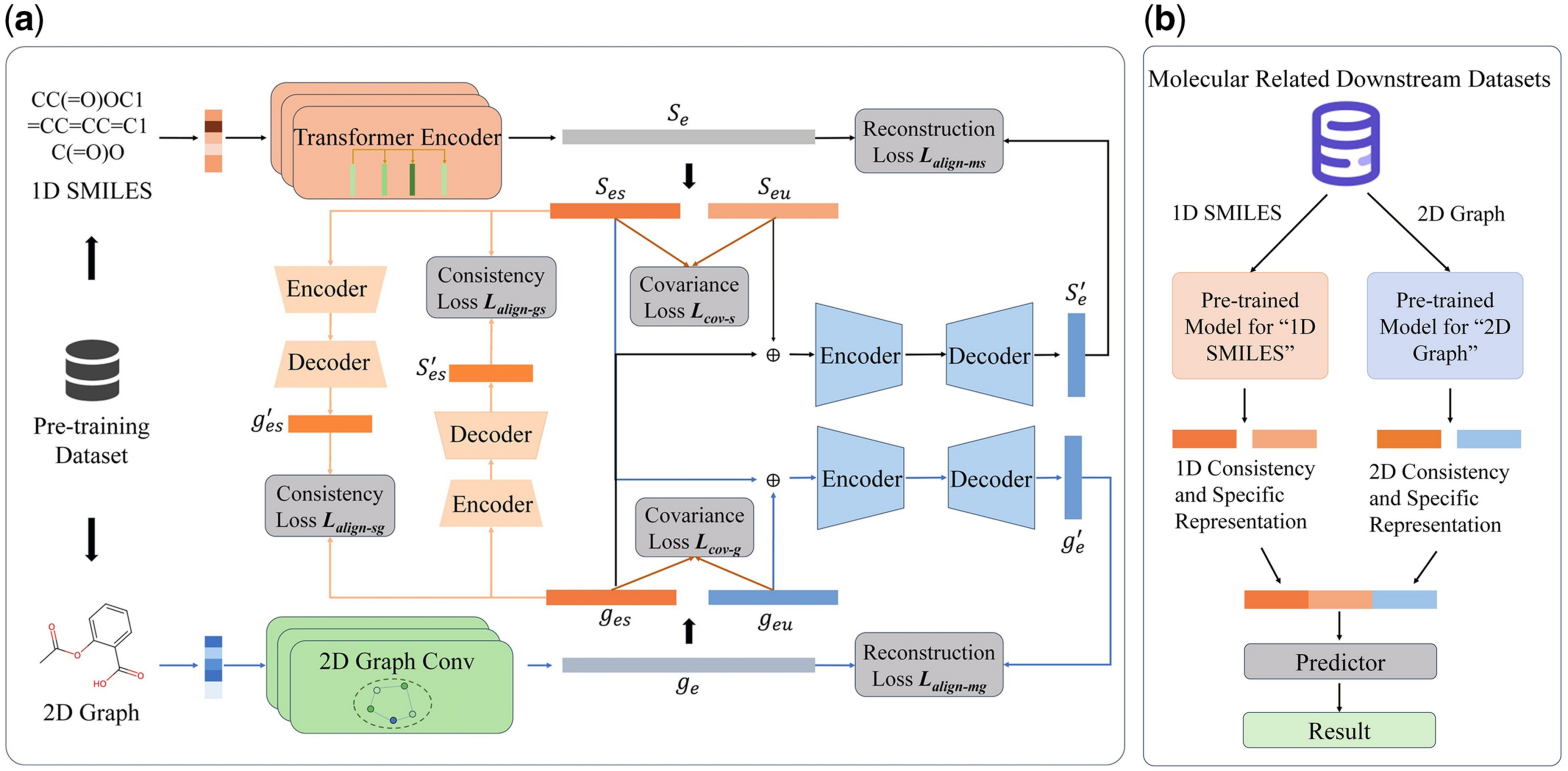
Flowchart of the MMPCS framework, illustrating its pretraining and fine-tuning stages. (a) During pretraining, MMPCS uses covariance and alignment loss to extract information from two distinct molecular views. (b) In fine-tuning, MMPCS leverages both SMILES and molecular graphs to enhance performance on downstream tasks.

This section provides a detailed description of the MMPCS. Figure 1 provides an overview of the model, which consists of two main stages: the pretraining stage and downstream task fine-tuning stage. In the pretraining stage, we first extract the molecular topology and SMILES sequence features. Subsequently, we introduce covariance loss to extract the consistency features and specific features of each view and align the consistency feature representations of the two views through consistency loss. In addition, to capture the holistic information of each view, we combine its specific information with the consistency information from the counterpart view to reconstruct the original features via an autoencoder. In the fine-tuning stage, to reduce the redundant information between views, we only concatenate the SMILES consistency representation with the view-specific representation from each view and pass the combined representation to the predictor for the final output.

#### 2.4.1 Molecular topological graph feature extraction

MMPCS uses a Graph Neural Network (GNN) for feature extraction from the molecular graph, $M_{g}$. Following Hu *et al.* (Hu *et al.* 2020), the Graph Isomorphism Network (GIN) is adopted as the backbone network. For a K-layer GIN, the molecular graph is initialized by incorporating atom-level and bond-level information. Specifically, the node features are initialized based on atom type $a_{t}$ and chirality $a_{c}$, while the edge features are initialized from bond type $b_{t}$ and bond direction $b_{d}$. All these feature representations are obtained through random initialization.


(8) $$h_{v}^{0}=W_{at}a_{t}+W_{ac}a_{c}$$

(9) $$e_{b}=W_{bt}b_{t}+W_{bd}b_{d}$$

where $W_{at},\;W_{ac},\;W_{bt},\;W_{bd}$ are learnable parameters; The $h_{v}^{0}$ represents the initial embedding of node v fed into the GNN; and $e_{b}$ represents the embedding of edge b. For node v at the k-th layer (1≤k ≤K), the output embedding $h_{v}^{k}$ of the GIN can be expressed by Equation (10):


(10) $$h_{v}^{k}=\mathrm{ML}P^{k}(h_{v}^{k-1},\;\mathrm{Agg}(\{h_{u}^{k-1}:u\in N(v)\},\;e_{b}))$$

here N(v) denotes the set of neighbors of node v. Here, the aggregation function Agg(⋅) is the sum function. To further extract the graph-level features $h_{G}$, a Readout(·) operation is performed on all node features, as shown in Equation (11):


(11) $$h_{G}=\mathrm{Readout}(\{h_{v}^{K}|v\in V_{g}\})$$

Readout(·) uses global average pooling. Finally, $h_{G}$ is fed into an MLP layer to better represent the holistic graph representation of the molecular graph, as shown in Equation (12):


(12) $$g_{e}=\mathrm{MLP}(h_{G})$$

where $g_{e}$ is the embedding of the 2D molecular topological graph.

#### 2.4.2 SMILES sequence feature extraction

$\mathrm{RoBERT}a_{\mathrm{base}}$ model (Liu *et al.* 1907) is used for feature extraction from the molecular SMILES sequence $M_{s}$, as shown in Equation (13):


(13) $$\mathrm{tokeniz}{ed}_{\mathrm{rep}}=\mathrm{tokenizer}(M_{s})$$

Here, tokenizer(·) is the tokenizer corresponding to $\mathrm{RoBERT}a_{\mathrm{base}}$, and $h_{s}$ is the encoded SMILES sequence.


(14) $$h_{s}=\mathrm{RoBERT}a_{\mathrm{base}}(\mathrm{tokenize}d_{\mathrm{rep}})$$

Finally, $h_{s}$ is fed into an MLP layer to learn a more expressive embedding for the SMILES sequence, as shown in Equation (15):


(15) $$S_{e}=\mathrm{MLP}(h_{s})$$

where $S_{e}$ is the embedding of the SMILES sequence.

#### 2.4.3 Consistency information extraction and alignment

Since multi-view data represent the same sample from different perspectives, they inherently share common (consistency) information, while also containing unique (specific) information. To disentangle these, covariance is used to separate the representations into consistency and view-specific information, thereby minimizing their mutual correlation. Finally, the consistency information across views is aligned to enhance representation learning.

**Extracting consistency information and specific information**. Covariance loss quantifies the correlation between two vectors. A positive value denotes a positive correlation between the two vectors, while a negative value indicates a negative correlation. Therefore, to fully decouple the consistency information from the specific information, we minimize their covariance loss, aiming for a value of zero which implies no linear relationship. Thus, the covariance loss between vectors x and y is given in Equation (16):


(16) $$\mathrm{CovLoss}(x,\;y)=\sum_{i=1}^{n}{\left(x_{i}-\bar{x}\right)}^{2}{\left(y_{i}-\bar{y}\right)}^{2}$$

The inter-view consistency information, denoted as $S_{es}$ and $g_{es}$, is taken from the first halves of the SMILES sequence embedding $S_{e}$ and the molecular graph embedding $g_{e}$, respectively. While the specific information, denoted as $S_{eu}$ and $g_{eu}$, is taken from their latter halves. Therefore, we have the following Equations (17) and (18):


(17) $$S_{e}=\mathrm{concat}(S_{es},\;S_{eu})$$

(18) $$g_{e}=\mathrm{concat}(g_{es},\;g_{eu})$$

where $S_{es}$ and $S_{eu}$ share the same dimensions, and similarly, so do $g_{es}$ and $g_{eu}$. Therefore, the covariance loss for each view is defined by Equations (19) and (20) as follows:


(19) $$L_{\mathrm{cov}-s}=\mathrm{CovLoss}(S_{es},\;S_{eu})$$

(20) $$L_{\mathrm{cov}-g}=\mathrm{CovLoss}(g_{es},\;g_{eu})$$

Finally, the total covariance loss $L_{\mathrm{cov}}$ is obtained:


(21) $$L_{\mathrm{cov}}=L_{\mathrm{cov}-s}+L_{\mathrm{cov}-g}$$

Algorithm 1: Pretraining Procedure of MMPCS

1: **Input:** Dataset *D* of paired molecule SMILES $M_{s}$ and 2D molecular graphs $M_{g}$.

2: **Initial:** molecular graph encoder *(GIN+MLP)*, molecular SMILES encoder *(RoBERTa + MLP)*, Consistency Projectors $P_{sg}$, $P_{gs}$, Molecule Graph and SMILES sequence feature reconstruction autoencoder MGEP, MSEP.

3: **for** *epoch *= 1 **to** *Pretrain_Epoch* **do** 

4:  **for each** batch$\;(M_{s},M_{g})$  **from**  D  **do** 

5:    /* Obtain 2D graph embeddings $g_{e}$ and SMILES embeddings $S_{e}$ from views $M_{g}$ and $M_{s}$ */

6:    Obtain the 2D graph embeddings $g_{e}$ [Equations (8)–(12)]

7:    Obtain the SMILES embeddings $S_{e}$ [Equations (13)–(15)]

8:    /* Split embeddings into consistency and specific parts */

9:    Obtain the consistency information $S_{es}$ and specific information $S_{eu}$ of SMILES view [Equation (17)]

10:  Obtain the consistency information $g_{es}$ and specific information $g_{eu}$ of 2D graph view [Equation (18)]

11:  Calculate Covariance Loss $L_{\mathrm{cov}}$ [Equations (19)–(21)]

12:   /* Align the consistency information */

13:  Predict graph consistency information $g_{es}^{'}$ from $S_{es}$ [Equations (22) and (23)]

14:  Predict SMILES consistency information $S_{es}^{'}$ from $g_{es}$ [Equations (24) and (25)]

15:  Calculate consistency information alignment losses $L_{\mathrm{align}-sg}$ and $L_{\mathrm{align}-gs}$ [Equations (27) and (28)]

16:  /* View feature reconstruction */

17:  Reconstruct the 2D graph embeddings based on $S_{e}$ and $g_{eu}$ [Equations (29) and (30)]

18:  Reconstruct the SMILES embeddings based on $g_{e}$ and $S_{eu}$ [Equations (31) and (32)]

19:  Calculate reconstruction loss $L_{\mathrm{align}-mg}$ and $L_{\mathrm{align}-ms}$ [Equations (33) and (34)]

20:  /* Calculate the overall alignment loss and the overall pretraining loss */

21:  Obtain the overall alignment loss $L_{\mathrm{align}}$ [Equation (35)]

22:  Calculate pretraining Loss L through Equation (36) and update parameters

23: **end for** 

24: **end for** 

25: **Return** Pretraining parameters

**Aligning the consistency information**. We introduce a consistency alignment mechanism that projects both views into a common embedding space using projection networks with identical architectures. This mechanism not only aligns consistency information bidirectionally between the two views but also supports cross-view reconstruction: the consistency information from one view, when combined with the specific information of another view, can reconstruct the complete representation of that other view. This design is motivated by the shared nature of consistency information and the complementary role of view-specific information.

Specifically, we use a SMILES-to-graph projector ($P_{sg}$) to predict the consistency information $g_{es}^{'}$ of the molecular graph from the consistency information $S_{es}$ of the SMILES, as shown in Equations (22) and (23):


(22) $$Z_{sg}=P_{sg}Enc(S_{es})$$

(23) $$g_{es}^{'}=P_{sg}Dec(Z_{sg})$$

where $Z_{sg}$ is obtained by encoder $P_{sg}Enc$. Then, $Z_{sg}$ is decoded by decoder $P_{sg}Dec$ to predict the consistency information $g_{es}^{'}$. Both the encoder and decoder each consist of a three-layer MLP. Symmetrically, we use a graph-to-SMILES projector ($P_{gs}$) to predict the SMILES consistency information $S_{es}^{'}$. This process is formulated in Equations (24) and (25):


(24) $$Z_{gs}=P_{gs}Enc(g_{es})$$

(25) $$S_{es}^{'}=P_{gs}Dec(Z_{gs})$$

where $Z_{gs}$ is obtained by encoder $SEP_{gs}Enc$. Then, $Z_{gs}$ is decoded by decoder $P_{gs}Dec$ to predict the consistency information $S_{es}^{'}$.

Based on the above alignment rationale, two consistency information alignment losses are defined: $L_{\mathrm{align}-sg}$ for predicting the graph view’s consistency information from that of the SMILES view, and $L_{\mathrm{align}-gs}$ for predicting the SMILES view’s consistency information from that of the graph view. Let x denote the ground-truth consistency vector, and $x^{'}$ represents the reconstructed prediction. The Mean Squared Error (MSE) used to calculate the consistency information alignment loss is expressed as Equation (26):


(26) $$\mathrm{MSE}(x^{'},\;x)=\frac{1}{n}\sum_{i=1}^{n}{\left(x_{i}^{'}-x_{i}\right)}^{2}$$

$L_{\mathrm{align}-sg}$ can be obtained using Equation (27):


(27) $$L_{\mathrm{align}-sg}=\mathrm{MSE}(g_{es}^{'},\;g_{es})$$

$L_{\mathrm{align}-gs}$ is defined by Equation (28):


(28) $$L_{\mathrm{align}-gs}=\mathrm{MSE}(S_{es}^{'},\;S_{es})$$

**View feature reconstruction.** As established, the shared consistency information enables cross-view reconstruction. Accordingly, the complete representation of each view is reconstructed by combining its specific information with the consistency information from the other view. To achieve this, we implement a Molecule Graph Embedding Projector (MGEP) and a Molecule SMILES Embedding Projector (MSEP), wherein both are designed as three-layer MLPs to mutually reconstruct the respective view representations and enhance model learning. The reconstruction of the molecular graph embedding is formally defined in Equations (29) and (30) as follows:


(29) $$Z_{mg}=\mathrm{MGE}P_{\mathrm{Enc}}(\mathrm{concat}(S_{es},\;g_{eu}))$$

(30) $$g_{e}^{'}=\mathrm{MGE}P_{\mathrm{Dec}}(Z_{mg})$$

Similarly, the reconstruction of the SMILES sequence embedding is defined in Equations (31) and (32) as follows:


(31) $$Z_{ms}=\mathrm{MSE}P_{\mathrm{Enc}}(\mathrm{concat}(g_{es},\;S_{eu}))$$

(32) $$S_{e}^{'}=\mathrm{MSE}P_{\mathrm{Dec}}(Z_{ms})$$

The reconstruction performance is assessed with two loss functions. The first measures the reconstruction loss for the molecular graph embedding as defined by Equation (33), while the second quantifies the loss for the SMILES sequence embedding reconstruction using Equation (34).


(33) $$L_{\mathrm{align}-mg}=\mathrm{MSE}(g_{e}^{'},\;g_{e})$$

(34) $$L_{\mathrm{align}-ms}=\mathrm{MSE}(S_{e}^{'},\;S_{e})$$

Thus, the total alignment loss $L_{\mathrm{align}}$ between the SMILES and 2D molecular topological graph views is computed as the sum of the four component losses, given by Equation (35):


(35) $$L_{\mathrm{align}}=L_{\mathrm{align}-sg}+L_{\mathrm{align}-gs}+L_{\mathrm{align}-mg}+L_{\mathrm{align}-ms}$$

The overall pretraining loss L is defined by Equation (36) as the weighted sum of $L_{\mathrm{cov}}$ and $L_{\mathrm{align}}$, where the coefficients α and β balance their respective contributions.


(36) $$L=\alpha L_{\mathrm{cov}}+\beta L_{\mathrm{align}}$$

The pseudocode summarizing the MMPCS pretraining process is presented in Algorithm 1.

## 3 Results

### 3.1 Empirical setting

To ensure a fair comparison, both MMPCS and the baseline methods were pretrained for the same number of epochs on identical datasets. For the downstream tasks, we adopted a consistent data-splitting strategy, partitioning each dataset into training, validation, and test sets in an 8:1:1 ratio. During fine-tuning, all prediction tasks were fine-tuned simultaneously, with each task using a dedicated projection head. The inputs for these tasks integrated both view-consistency and view-specific information. The key hyperparameters and their configurations for both pretraining and fine-tuning stages are detailed in Table 4.

**Table 4:** Hyperparameters of the MMPCS model.

Hyperparameters	Setting
Pretrain epoch	1
Pretrain batch size	32
Learning rate	0.00005
Dim of final SMILES/graph embedding	256
Num of GIN conv layer	3
Coefficient of loss α, β	[1, 1]
Fine-tuning epoch	100
Fine-tuning batch size	64

### 3.2 Comparison of MMPCS with baseline methods for property prediction

To evaluate the effectiveness of our pretraining model, it was benchmarked against 16 classic and state-of-the-art methods which are categorized into three groups:

- Sequence-based pretraining methods: SMILES-Transformer (Honda *et al.* 2019).
- Graph-based pretraining methods: GraphCL (You *et al.* 2020), JOAO (You *et al.* 2021), JOAOv2 (You *et al.* 2021), GraphLoG (Xu *et al.* 2021), Lp-Info (You *et al.* 2022), SimGRACE (Xia *et al.* 2022), AD-GCL (Suresh *et al.* 2021), MGSSL (Zhang *et al.* 2021), GraphMAE (Hou *et al.* 2022), MolCLR (Wang *et al.* 2022), Mole-BERT (Xia *et al.* 2023b).
- Multi-view pretraining methods: GraphMVP (Liu *et al.* 2022), 3DInfomax (Stärk *et al.* 2022), MEMO (Zhu *et al.* 2022), and FragCL (Kim *et al.* 2023).

The performance comparison of various methods on molecular attribute classification tasks is summarized in Table 5. MMPCS achieved an average ROC-AUC of 86.35% across seven benchmark datasets, surpassing existing methods such as MolCLR and MGSSL. This notable improvement highlights the efficacy of our pre-training framework. Furthermore, MMPCS exhibited strong performance on toxicity- and side effect-related datasets, including Tox21, ToxCast, SIDER, and ClinTox. On ToxCast, it attained a ROC-AUC of 86.81%, representing a 9.7% gain over the second-best method (SMILES-Transformer). Similarly, it achieved 82.55% on SIDER and 98.76% on ClinTox, indicating substantial improvements over baseline models. These gains can be attributed to the MMPCS’s pre-training framework that simultaneously: (i) suppresses irrelevant perturbations, (ii) compresses redundant features, and (iii) retains critical structural information. This capability is particularly important for toxicity prediction tasks that are highly sensitive to specific substructures such as nitro groups, halogens, and conjugated systems (Kazius *et al.* 2005, Hao *et al.* 2019). The comparatively weaker performance of perturbation-driven contrastive graph pretraining methods (such as GraphCL, JOAO, and JOAOv2) further supports our hypothesis that excessive structural perturbations may hinder the preservation of chemically critical substructures during molecular representation learning. On property prediction tasks across the BBBP, Estrogen, and MetStab datasets, MMPCS delivered strong overall results. It achieved top performance on the BBBP and Estrogen datasets and ranked second on MetStab. Notably, despite MEMO leveraging four molecular views, MMPCS surpassed it with only two views. The experimental outcomes collectively demonstrate that MMPCS effectively integrates multi-modal molecular information to produce comprehensive and discriminative molecular representations.

**Table 5:** Classification results (ROC-AUC, %) on molecular property prediction tasks.^a^

Category	Dataset	BBBP	Tox21	ToxCast	SIDER	ClinTox	BACE	Estrogen	MetStab	Avg
**Sequence-Based**	SMILES-Transformer	52.50	62.66	77.11	79.89	92.59	62.48	57.59	74.84	69.95
**Graph-Based**	GraphCL	70.05	58.17	50.58	54.83	95.25	78.1	85.08	77.57	71.20
	JOAO	68.99	50	50.26	53.47	92.25	77.9	88.76	83.46	70.63
	JOAOv2	71.76	55.75	50.76	53.74	92.9	72.03	91.08	77.43	70.68
	AD-GCL	69.98	50.52	51.33	52.93	90.19	75.98	76.98	74.91	67.85
	GraphLoG	68.95	62.55	50.51	48.69	95.97	76.09	75.21	72.82	68.84
	MGSSL	71.41	60.38	49.19	57.36	92.38	81.91	80.16	88.67	72.68
	Lp-Info	66.61	55.06	50.61	51.17	95.79	73.32	75.49	75.66	67.96
	SimGrace	63.14	52.4	50.02	53.26	92.68	74.3	83.1	79.33	68.52
	GraphMAE	68.8	55.08	51.27	52.75	95.33	76.66	85.97	81.21	70.88
	MolCLR	71.66	79.45	72.8	59.62	49.94	78.00	94.16	79.69	73.16
	Mole-BERT	69.26	57.25	49.59	50.18	95.49	75.51	90.95	79.98	71.02
**Multi-View**	GraphMVP*	68.5	74.5	62.7	62.3	79.0	76.8	-	-	-
	3DInfomax*	69.1	74.46	64.41	53.37	59.43	79.42	-	-	-
	FragCL*	71.4	75.2	65.1	61	95.2	82.3	-	-	-
	MEMO*	71.6	76.7	64.9	61.2	81.6	**82.6**	-	-	-
	MMPCS (ours)	**73.41**	**81.33**	**86.81**	**82.55**	**98.76**	80.16	**95.14**	**92.65**	**86.35**

a Higher ROC-AUC values indicate better performance. “Avg” denotes the average result across all datasets. The best result for each dataset is highlighted in bold, and the second-best result is underlined. An asterisk (*) indicates that the experimental results were sourced from the original paper.

To further evaluate the generalizability of MMPCS, it was compared with baseline models on five downstream regression tasks. As summarized in Table 6, MMPCS achieved the best or second-best results on the four out of the five datasets and maintained the highest average performance, with particularly notable improvement on the ESOL dataset. These results demonstrate the competitiveness and robustness of MMPCS in regression settings. It is worth noting that multi-view baselines failed to achieve comparable performance, implying that the effective capture of view-specific information is crucial for regression tasks. Moreover, MMPCS outperformed all two-view models and surpassed even the four-view model MEMO by a significant margin.

**Table 6:** Regression results (RMSE) on molecular property prediction tasks.^a^

Category	Dataset	ESOL	Lipo	FreeSolv	Malaria	CEP	Avg
Sequence-Based	SMILES-Transformer	2.095	1.121	3.790	1.176	2.539	2.144
Graph-Based	GraphCL	0.989	0.726	1.697	0.998	1.302	1.142
	JOAO	0.807	0.705	**1.472**	1.035	1.466	1.097
	JOAOv2	0.809	0.698	1.637	1.046	1.477	1.133
	AD-GCL	0.936	0.758	1.793	0.977	1.646	1.222
	GraphLoG	0.927	0.714	1.956	0.984	1.337	1.184
	MGSSL	0.766	0.725	1.595	**0.956**	1.292	1.067
	Lp-Info	1.191	0.709	1.885	0.977	1.320	1.216
	SimGRACE	1.365	0.838	1.861	0.997	1.363	1.285
	GraphMAE	1.007	0.701	1.676	0.986	1.289	1.132
	MolCLR	0.861	**0.623**	1.804	0.998	**1.004**	1.058
	Mole-BERT	0.838	0.679	1.866	0.982	1.304	1.134
Multi-View	GraphMVP*	1.091	0.718	-	1.114	1.236	-
	3DInfomax*	0.894	0.695	2.337	-	-	-
	MEMO*	0.984	0.707	-	1.093	1.101	-
	MMPCS (ours)	**0.595**	0.649	1.473	0.999	1.018	**0.946**

a Lower RMSE values indicate better performance. “Avg” denotes the average result across all datasets. The best result for each dataset is highlighted in bold, and the second-best result is underlined. An asterisk (*) indicates that the experimental results were sourced from the original paper.

### 3.3 MMPCS for other drug-related tasks

To evaluate the effectiveness of molecular representations in drug-related tasks, we leveraged the pre-trained MMPCS model to generate representations for the CDR and DTA prediction tasks. For CDR prediction, the baseline methods included DeepTTA (Jiang *et al.* 2022), DeepCDR (Liu *et al.* 2020), tCNNs (Liu *et al.* 2019), CDRscan (Chang *et al.* 2018), and MOLI (Sharifi-Noghabi *et al.* 2019). For DTA prediction, the compared methods were GraphDTA(Nguyen *et al.* 2021) WideDTA (Öztürk *et al.* 2019), DeepDTA (Öztürk *et al.* 2018), SimBoost (He *et al.* 2017), and KronRLS (Cichonska *et al.* 2018). In these experiments, the original drug-encoding modules of the baseline models were substituted with representations generated by MMPCS. Specifically, the drug-processing component of DeepTTA was substituted in the CDR prediction task, and similarly, that of GraphDTA was replaced in the DTA prediction task. To maintain architectural consistency, the MMPCS representations were adapted to the required hidden dimensions via a lightweight linear projection layer, while the protein-encoding components of both DeepTTA and GraphDTA remained unchanged in structure and parameters.

As summarized in Tables 7 and 8, MMPCS achieved the best performance across all evaluation metrics, surpassing all baseline methods. These results demonstrate that the molecular representations learned by MMPCS possess strong generalization capabilities and can be effectively transferred to improve performance in key drug discovery tasks.

**Table 7:** Performance of various methods on the CDR task.^a^

Methods	PCC↑	SCC↑
MoLI	0.813	0.782
CDRscan	0.871	0.852
tCNNs	0.910	0.889
DeepCDR	0.923	0.898
DeepTTA	0.941	0.914
MMPCS	**0.946**	**0.933**

a Baseline results are sourced from DeepTTA. The best score for each metric is highlighted in bold.

**Table 8:** Performance of various methods on the DTA task.^a^

Methods	CI↑	MSE↓
KronRLS	0.782	0.411
SimBoost	0.836	0.222
DeepDTA	0.865	0.194
WideDTA	0.875	0.179
GraphDTA	0.882	0.174
MMPCS	**0.882**	**0.150**

a Baseline results are sourced from GraphDTA. The best score for each metric is highlighted in bold.

**Figure 2:**
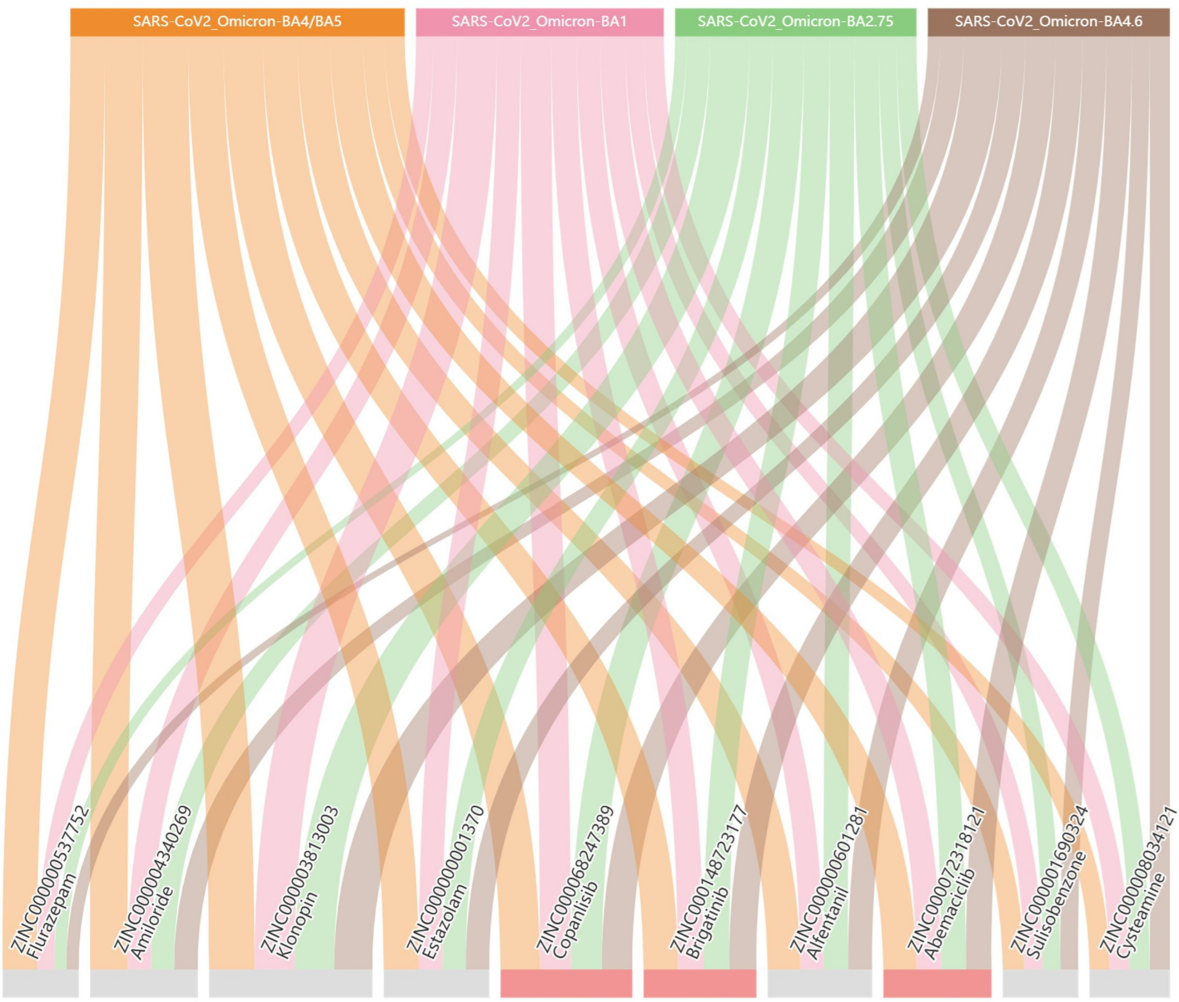
Sankey diagram visualizes the predicted drug–target affinities between four SARS-CoV-2 Omicron variant proteins (top) and the top 10 FDA-approved drugs (bottom). The width of each ribbon represents the predicted affinity strength, where a thicker ribbon denotes a stronger affinity.

To further validate its practical utility, MMPCS was applied to an important real-world task, antiviral drug repurposing for the SARS-CoV-2 Omicron variant. We selected four major variants (BA.2.75, BA.4/BA.5, BA.4.6, BA.1) and screened 1615 FDA-approved drugs from the ZINC database as potential inhibitors. GraphDTA, fine-tuned on KIBA dataset, incorporated MMPCS for drug processing to predict drug-RBD affinity. Based on their predicted binding affinity against four Omicron variants, the ten top-ranked candidates were selected for further analysis. As illustrated in Fig. 2, several compounds with potential antiviral activity were identified, meriting further validation. Among them, three drugs highlighted in red have been documented to inhibit SARS-CoV-2. Specifically, Abemaciclib (ZINC000072318121) effectively inhibits SARS-CoV-2 replication in vitro, reduces cytopathic effects, and demonstrates favorable antiviral efficacy and safety in animal models(Wang *et al.* 2021). Copanlisib (ZINC000068247389) mimics the binding mode of VIR251, showing high affinity and potential as an inhibitor. It significantly inhibits Omicron pseudo viruses by reducing viral entry via endocytosis, highlighting its promise for infection prevention (Kuzikov *et al.* 2022). Brigatinib (ZINC000148723177), a multi-target tyrosine kinase inhibitor, potently suppresses SARS-CoV-2 spread. In vivo studies have demonstrated that oral administration of Brigatinib can reduce viral load and alleviate lung inflammation in mice, though further clinical trials are still required to confirm its safety and efficacy in humans (Shin *et al.* 2024). Collectively, these experimental findings further corroborate the effectiveness of the MMPCS-based drug screening approach in identifying potential antiviral candidate compounds.

### 3.4 Ablation study

In this section, we conduct ablation studies on seven representative datasets from MoleculeNet to evaluate the contribution of each component in the MMPCS framework. The selected datasets cover diverse classification tasks (BBBP, SIDER, ClinTox and Estrogen) and regression tasks (ESOL and FreeSolv), encompassing a wide range of molecular properties and task characteristics. In the following subsections, we systematically evaluated the impact of network architecture design and decorrelation loss functions to validate the effectiveness and rationality of each MMPCS component.

#### 3.4.1 The influence of model components

In this section, we conducted ablation studies from three perspectives: loss function design, view-specific information utilization and pretraining usage. First, to investigate the necessity of combining both loss functions, we constructed two model variants: one using only the consistency alignment loss (${\text{MMPCS}}_{w/o\;\text{cov}}$) and the other using only the covariance loss (${\text{MMPCS}}_{w/o\;\text{align}}$). Second, to evaluate the contribution of view-specific information, we fine-tuned the model using solely the SMILES sequence (${\text{MMPCS}}_{\text{SMILES}}$) or the molecular graph (${\text{MMPCS}}_{\text{graph}}$). Fine-tuning with a single view is equivalent to discarding the specific information from the other view during inference, which may limit the representational capacity of the model. Finally, to assess the importance of pretraining, we evaluated a non-pretrained variant (${\text{MMPCS}}_{w/o\;\text{pre}}$).

As shown in Table 9, the ablation studies revealed several key findings. First, MMPCS consistently outperformed ${\text{MMPCS}}_{w/o\;\text{cov}}$ and ${\text{MMPCS}}_{w/o\;\text{align}}$ across most tasks, especially on BBBP, ESOL, and FreeSolv. This demonstrates that jointly optimizing both losses functions is essential for effectively balancing shared and view-specific representation learning. Second, fine-tuning with both views outperformed the single-view variants, highlighting the complementary nature of SMILES and graph representations. On SIDER, where inter-view consistency information plays a dominant role, the performance gap between single-view and dual-view models is small. In contrast, for tasks such as SIDER and ClinTox, which are more sensitive to local molecule structures, the graph provides critical view-specific information. For ESOL, SMILES contributes more to prediction, while on FreeSolv, the graph view is more informative. These observations collectively confirm the task-dependent complementarity of the two molecular modalities. Third, the non-pretrained variant ${\text{MMPCS}}_{w/o\;\text{pre}}$ exhibits noticeable performance degradation across all datasets. For example, on BBBP, MMPCS achieves a ROC-AUC of 73.41, while ${\text{MMPCS}}_{w/o\;\text{pre}}$ drops to 70.02. A similar trend is observed on ESOL, where the RMSE of ${\text{MMPCS}}_{w/o\;\text{pre}}$ increases by 0.072 compared to the fully pretrained model. These results indicate that pretraining process enables the model to learn transferable molecular knowledge that benefits downstream tasks.

**Table 9:** Ablation study evaluating MMPCS under different variants on representative datasets.

Dataset	BBBP	SIDER	Estrogen	ClinTox	ESOL	FreeSolv
		
	ROC-AUC↑				RMSE↓	
${\text{MMPCS}\;}_{w/o\;\text{align}}$	68.91	81.92	94.83	95.85	0.617	2.315
${\text{MMPCS}}_{w/o\;\text{cov}}$	67.23	82.53	94.97	98.72	0.802	1.860
${\text{MMPCS}}_{\text{SMILES}}$	60.08	80.68	62.23	91.30	0.721	4.009
${\text{MMPCS}}_{\text{graph}}$	65.84	82.41	94.11	96.48	0.928	1.788
${\text{MMPCS}}_{w/o\;\text{pre}}$	70.02	82.12	94.96	98.53	0.667	1.489
MMPCS	**73.41** ^a^	**82.55**	**95.14**	**98.76**	**0.595**	**1.473**

a The best score for each metric is highlighted in bold.

#### 3.4.2 The influence of decorrelation loss functions

To systematically assess the efficacy of the covariance loss in MMPCS for representation decoupling, we benchmarked MMPCS against three established decorrelation approaches: Orthogonality Loss (OL), Hilbert-Schmidt Independence Criterion (HSIC), and Mutual Information Neural Estimation (MINE). Across all comparisons, only the covariance loss was substituted, while all other settings remained unchanged.

As shown in Table 10, the covariance loss achieved either superior or highly competitive performance across all five benchmark datasets, achieving the highest average performance among all evaluated methods. This validates its effective balance between decorrelation effectiveness and training stability, establishing it as a practical and reliable choice for multi-view molecular representation learning in MMPCS.

**Table 10:** Comparison of different decorrelation loss functions on representative datasets.

Dataset	BBBP	SIDER	Estrogen	ESOL	FreeSolv
		
	ROC-AUC↑			RMSE↓	
${\text{MMPCS}}_{\text{OL}}$	70.59	82.21	94.52	0.648	1.961
${\text{MMPCS}}_{\text{HSIC}}$	68.40	82.17	94.73	0.612	1.939
${\text{MMPCS}}_{\text{MINE}}$	70.87	81.69	88.11	1.288	2.128
MMPCS	**73.41** ^a^	**82.55**	**95.14**	**0.595**	**1.473**

a The best score for each metric is highlighted in bold.

### 3.5 The impact of hyperparameters

To further investigate the influence of hyperparameters on model performance, we conducted a sensitivity analysis on the weighting coefficients of the two primary loss components: the covariance loss (controlled by the α) and the consistency alignment loss (controlled by β). In this study, α was fixed at 1, while β was varied across the set {0.1, 0.2, 0.3, 0.5, 1, 2, 3, 5, 10}. The performance of MMPCS under these loss weight configurations was evaluated on five representative datasets.

**Figure 3:**
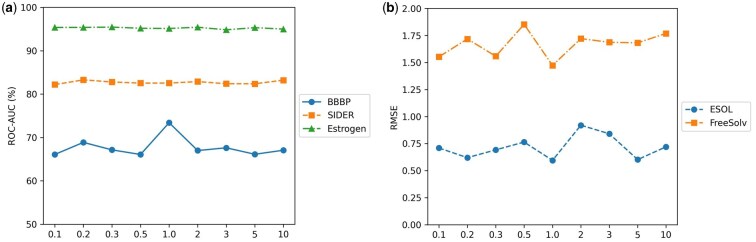
Performance trends of MMPCS under varying loss weight β (with α fixed at 1.0) for (a) classification and (b) regression tasks.

The performance trends under varying β (with α fixed at 1.0) are shown in Fig. 3. A consistent optimum is observed at β=1.0 across all tasks, with regression achieving its lowest RMSE and classification tasks their highest scores. This indicates that equally weighting the consistency and covariance losses (i.e. β=1.0) creates an optimal balance, allowing the model to leverage both objectives effectively and thus achieve the best overall performance.

### 3.6 Sensitivity analysis of GIN layers

**Figure 4:**
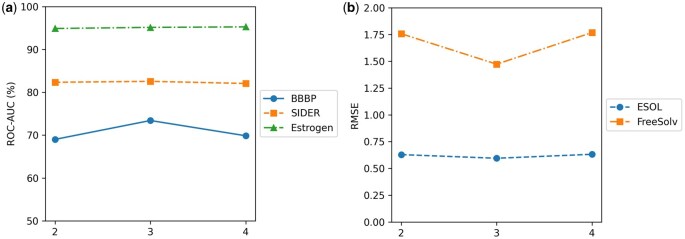
Effect of GIN network depth on model performance across (a) classification and (b) regression datasets.

To investigate the effect of GIN network depth on model performance, we evaluated architectures with 2, 3, and 4 layers across several representative datasets, including BBBP, SIDER, and Estrogen for classification, and ESOL and FreeSolv for regression. As shown in Fig. 4, the model with three GIN layers achieves the best or near-best performance across most datasets. In classification tasks, the three-layer GIN attains a ROC-AUC of 73.41% on BBBP, substantially outperforming both the two- and four-layer variants, while also maintains stable superiority on SIDER and Estrogen. In regression tasks, it achieves the lowest RMSE on both ESOL and FreeSolv, demonstrating its stronger numerical approximation capability. Overall, reducing the depth to two layers limits the model’s expressive power, whereas increasing it to four layers results in performance degradation, likely due to over-smoothing and training instability. These findings collectively indicate that the three-layer GIN provides an optimal balance between representational capacity and model stability.

### 3.7 Visualization

**Figure 5:**
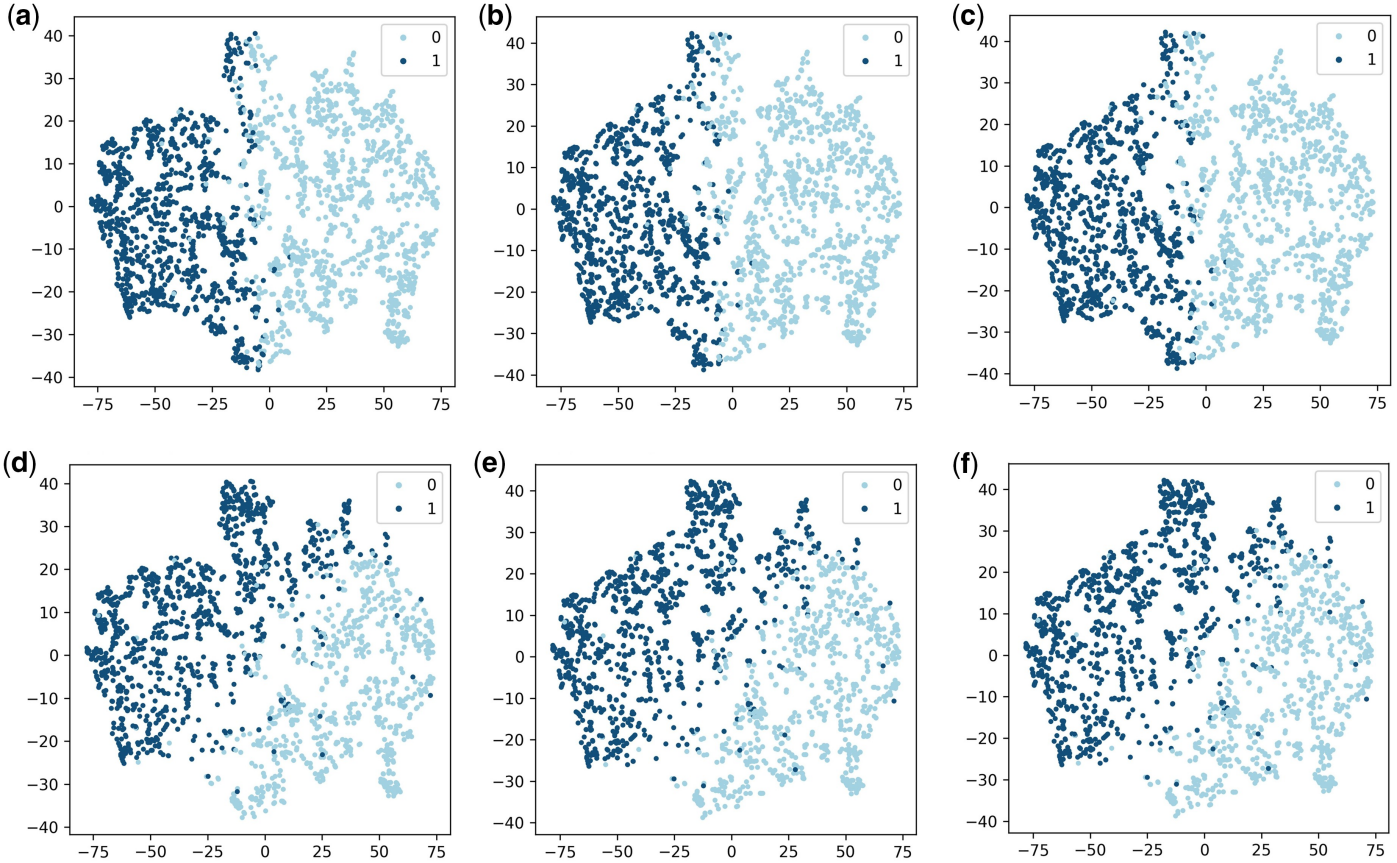
t-SNE visualization of molecular representations fine-tuned on the Estrogen dataset, which contains two binary classification tasks (α and β). The first row (a–c) corresponds to the α task, and the second row (d–f) to the β task. (a) and (d): SMILES embedding concatenated with the view-specific information of the molecular graph ($S_{e}∥g_{eu}$); (b) and (e): view-specific information of the SMILES sequence concatenated with the embedding of the molecular graph ($S_{eu}∥g_{e}$); (c) and (f): direct concatenation of the SMILES and molecular graph embeddings ($S_{e}∥g_{e}$).

For the downstream tasks, MMPCS utilizes inter-view consistency and view-specific information as inputs. Unlike a simple concatenating of the two view embeddings, MMPCS reduces the feature dimensionality by 25%. This efficiency gain is achieved by avoiding the duplication of inter-view consistency information. To demonstrate the effectiveness of this approach, t-SNE visualizations were generated for all molecules in the Estrogen dataset using three distinct representations: (1) $S_{e}∥g_{eu}$: SMILES embedding concatenated with the view-specific information of the molecular graph; (2) $S_{eu}∥g_{e}$: the view-specific information of the SMILES concatenated with the embedding of the molecular graph, and (3) $S_{e}∥g_{e}$: direct concatenation of the SMILES and molecular graph embeddings. The Estrogen dataset supports two binary-classification tasks, α and β, which are displayed in the first and second rows of Fig. 5, respectively, with the legend indicating the ground-truth labels for each task.

The t-SNE visualizations reveal two key findings. First, the strong similarity between $S_{e}∥g_{eu}$ (Fig. 5a) and $S_{eu}∥g_{e}$ (Fig. 5b) indicates that MMPCS effectively extracts consistent inter-view information, regardless of which view provides the base embedding. Similar trends are observed in Fig. 5d and e. Additionally, $S_{e}∥g_{eu}$ (Fig. 5a) closely matches $S_{e}∥g_{e}$ (Fig. 5c), confirming our method preserves information while reducing input dimensionality, as further supported by Fig. 5d and f.

The nearly identical DBI values across the three different connection methods, specifically 0.72, 0.74, and 0.74 for task α in Fig. 5(a)–(c), and 0.89, 0.91, and 0.91 for task β in Fig. 5(d)–(f), confirm their comparable clustering performance, thereby validating the reliability of our method. This is visually supported by the high similarity between the first two columns in Fig. 5, indicating that SMILES and molecular graph embeddings encode nearly identical consistency information. Furthermore, the close resemblance between these and the third column demonstrates that MMPCS achieves significant dimensionality re duction without losing critical information.

## 4 Conclusion

In this study, we proposed MMPCS, a multi-view pretraining framework that effectively captures both inter-view consistency and view-specific information without relying on data augmentation techniques. This design avoids the risk of excessively altering molecular properties through data augmentation, which could otherwise lead to biased representations. Furthermore, by eschewing chemistry-specific augmentation strategies and rule-based substructure design, MMPCS minimizes its dependence on manually crafted domain knowledge, while still leveraging general molecular priors learned through data-driven pretraining. Extensive experiments demonstrated that MMPCS achieves superior performance, outperforming existing methods on average across eight classification datasets and five regression tasks. It also achieves state-of-the-art performance in DTA and CDR prediction, and demonstrates promising potential for COVID-19 drug repositioning. Ablation studies confirm the necessity of combining both loss functions and highlights the importance of incorporating view-specific information. Visualization results further validate the effectiveness of our approach in generating compact yet informative representations for downstream tasks. Looking forward, we plan to extend MMPCS by incorporating additional molecular views during pretraining. A current limitation is the requirement for consistency and view-specific information to share identical dimensions within the same view. Future work will focus on developing more flexible mechanisms to acquire this information adaptively.

## Data Availability

The code and data for reproducing the MMPCS framework are publicly available on GitHub (https://github.com/xmubiocode/MMPCS) and archived on Zenodo (https://doi.org/10.5281/zenodo.18182748).
